# Comparative transcriptome analysis reveals sesquiterpenoid biosynthesis among 1-, 2- and 3-year old *Atractylodes chinensis*

**DOI:** 10.1186/s12870-021-03131-1

**Published:** 2021-07-27

**Authors:** Jianhua Zhao, Chengzhen Sun, Fengyu Shi, Shanshan Ma, Jinshuang Zheng, Xin Du, Liping Zhang

**Affiliations:** Hebei Key Laboratory of Crop Stress Biology (in Preparation), Hebei Normal University of Science & Technology, Qinhuangdao, 066004 Hebei China

**Keywords:** *Atractylodes chinensis* (DC.) Koids., Transcriptome, Differentially expressed genes, Sesquiterpenoid, qRT-PCR

## Abstract

**Background:**

*Atractylodes chinensis* (DC.) Koidz is a well-known medicinal plant containing the major bioactive compound, atractylodin, a sesquiterpenoid. High-performance liquid chromatography (HPLC) analysis demonstrated that atractylodin was most abundant in 3-year old *A. chinensis* rhizome, compared with those from 1- and 2-year old rhizomes, however, the molecular mechanisms underlying accumulation of atractylodin in rhizomes are poorly understood.

**Results:**

In this study, we characterized the transcriptomes from rhizomes of 1-, 2- and 3-year old (Y1, Y2 and Y3, respectively) *A. chinensis*, to identify differentially expressed genes (DEGs). We identified 240, 169 and 131 unigenes encoding the enzyme genes in the mevalonate (MVA), methylerythritol phosphate (MEP), sesquiterpenoid and triterpenoid biosynthetic pathways, respectively. To confirm the reliability of the RNA sequencing analysis, eleven key gene encoding factors involved in the sesquiterpenoid and triterpenoid biosynthetic pathway, as well as in pigment, amino acid, hormone and transcription factor functions, were selected for quantitative real time PCR (qRT-PCR) analysis. The results demonstrated similar expression patterns to those determined by RNA sequencing, with a Pearson’s correlation coefficient of 0.9 between qRT-PCR and RNA-seq data. Differential gene expression analysis of rhizomes from different ages revealed 52 genes related to sesquiterpenoid and triterpenoid biosynthesis. Among these, seven DEGs were identified in Y1 vs Y2, Y1 vs Y3 and Y2 vs Y3, of which five encoded four key enzymes, squalene/phytoene synthase (SS), squalene-hopene cyclase (SHC), squalene epoxidase (SE) and dammarenediol II synthase (DS). These four enzymes directly related to squalene biosynthesis and subsequent catalytic action. To validate the result of these seven DEGs, qRT-PCR was performed and indicated most of them displayed lower relative expression in 3-year old rhizome, similar to transcriptomic analysis.

**Conclusion:**

The enzymes SS, SHC, SE and DS down-regulated expression in 3-year old rhizome. This data corresponded to the higher content of sesquiterpenoid in 3-year old rhizome, and confirmed by qRT-PCR. The results of comparative transcriptome analysis and identified key enzyme genes laid a solid foundation for investigation of production sesquiterpenoid in *A. chinensis*.

**Supplementary Information:**

The online version contains supplementary material available at 10.1186/s12870-021-03131-1.

## Background

*Atractylodes lancea* and *Atractylodes chinensis* (typically referred to as “Mao Cang Zhu” and “Bei Cang Zhu” in Chinese), together constitute the rhizome atractylodes, and belong to the Asteraceae family. The rhizome atractylodes are widely used in East Asia, and have great economic and medicinal value. *A. lancea* is currently on the verge of extinction, therefore, *A. chinensis* is the main source of the rhizome atractylodes that widely distributed in most areas of Northern China. The main bioactive compounds in *A. chinensis* rhizome are used to treat digestive disorders, rheumatic diseases and night blindness [6]. Modern pharmacological studies have demonstrated that *A. chinensis* also has anti-inflammatory, anti-bacterial [11, 20] and antitumor [13] properties. Although the sesquiterpenoid components of *A. chinensis* rhizome have important pharmacological activities, the molecular mechanisms underlying accumulation of bioactive sesquiterpenoids are poorly understood. In plants, sesquiterpenoids are generally synthesized via MVA and MEP biosynthetic pathways.

Natural populations of *A. chinensis* currently being rapidly depleted, due to heavy use and the weak reproductive capacity of perennial herbs. Thus, artificial cultivation is urgently needed to protect the natural populations and ensure sustainable utilization. A crucial question is how to ensure, or even improve, rhizome atractylodes quality, in terms of sesquiterpenoid content. Although the phytochemistry [11, 33], pharmacology [5, 14, 20, 24] and cultivation [27, 32, 37, 38] of *A. chinensis* have been studied, the molecular mechanisms underlying their accumulation of bioactive compounds remains unclear, largely due to a lack of genomic and transcriptomic data.

Transcriptome analysis is an effective approach for analysis of secondary metabolites biosynthesis, and has been used to determine the functions of genes in medicinal plants, including Danshen (*Salvia miltiorrhiza*) [34], Renshen (*Panax ginseng*) [4], Sanqi (*Panax notoginseng*) [18] and Yunnan chonglou (*Paris polyphylla* var. *yunnanensis*) [9], among others. Recently, understanding of the molecular processes involved in sesquiterpenoid biosynthesis has improved, with various genes involved in this biosynthetic pathway investigated by transcriptome analysis in the genus, *Atractylodes* [2, 12, 36]. Sesquiterpenoids are the main bioactive components in the rhizomes of *A. lancea* and *A. chinensis*; however, there are differences between them in the composition and content of sesquiterpenoids. In addition, the content of bioactive components in perennial medicinal herbs is influenced by the year of cultivation [1, 16, 29]. To date, one study has reported the transcriptome profiles of 3-year old *A. chinensis* rhizome [36]; however, there are no data regarding the molecular mechanism involved in the relationship between sesquiterpenoid accumulation and year of cultivation. Elucidating factors involved in the biosynthesis and accumulation of bioactive components and identifying key enzyme genes in the biosynthetic pathway will be important steps toward improvements in sesquiterpenoid production.

Here, rhizomes from 1-, 2- and 3-year old *A. chinensis* were subjected to high throughput transcriptome sequencing, enabling us to characterize the transcriptomes and differential expression profiles of *A. chinensis* rhizomes cultivated for different ages, to profile differentially expressed genes (DEGs) among rhizomes from different years of cultivation, and to identify DEGs related to biosynthesis and accumulation of sesquiterpenoids. Discovering the key enzyme genes in the sesquiterpenoid biosynthetic pathway is necessary to improve atractylodin production. This study could provide insights into the relationship between changes in atrctylodin content and year of cultivation, and contribute to uncovering the underlying molecular mechanisms in *A. chinensis*.

## Materials and methods

### Plant materials

*A. chinensis* seeds were collected from cultivation base of Qinhuangdao Tongsheng Pharmaceutical Co., Ltd, Qinhuangdao City, Hebei Province, China. To ensure a similar physical environment, seeds were sown separately in 2016, 2017 and 2018, in the same open field at the Hebei Normal University of Science & Technology. No permission is required to collect wild resources of *A. chinensis*. *A. chinensis* rhizome, which is the part of the plant used in medicinal preparations, serves as a store for photosynthetic products and bioactive compounds. For use as a medicine, *A. chinensis* is optimally harvested the 3rd to 4th rhizomes during late October to early November in Hebei province, therefore, 1-, 2- and 3-year old rhizomes with 3 biological replicates (15 rhizomes for each biological replicates) were collected in early November 2018 (as seen in Fig. 7). After collection, rhizomes were cleaned in running water, and divided into two groups. One group was dried at 60 °C for atractylodin extraction and HPLC analysis. The other group was immediately frozen in liquid nitrogen and stored at -80 °C prior to RNA extraction and sequencing.

### Atractylodin extraction and HPLC analysis

The dried rhizomes were and then ground into a powder, 0.2 g of which was immersed in 50 mL methanol (purity ≥ 99.9%, Grade/Application information: ACS. Reag. Ph Eur, CSA-No. 67–56-1) and ultrasonically extracted (Power 250 W, Frequency 40 kHz) for 1 h. Next, 1 mL of the supernatant was collected and passed through a 0.22 μm microporous filter membrane (JTSF0311, Tianjin Jinteng Experiment Equipment Co. Ltd.).

Determination of atractylodin content in 1-, 2- and 3-year old rhizomes was conducted using a Thermo Fisher UltiMate 3000 UPLC system, equipped with a Uv–vis detector, on C18 Column (4.6 × 250 mm, 5 μm, Thermo Fisher). The mobile phase was methanol:water (79:21), with a flow rate of 1.0 mL∙min^−1^. The HPLC chromatogram was monitored at 340 nm, and the column temperature was set at 30 °C.

The standard atractylodin (number 111924–201,806, gbw114.com, China) solution of 20 μg∙ml^−1^ was prepared with methanol, then diluted to 1 µg∙ml^−1^, 2 µg∙ml^−1^, 5 µg∙ml^−1^and 10 µg∙ml^−1^, separately. According to the relationship between standard atractylodin content (y) and its cover area (x), the standard curve was obtained y = 4.2254 x (R^2^ = 0.9999). Atractylodin content was determined by standard curve. The content of atractylodin (%) = y * 50 mL (methanol) / 0.2 g (rhizome powder). The mean values of three biological replicates were calculated. Statistical significance of atractylodin contents among 1-, 2- and 3-year old rhizomes was analyzed by DPS (version 14.5).

### RNA sequencing and functional annotation of unigenes

To extract total RNA, three biological replicates of rhizomes from 1-, 2- and 3-year old *A. chinensis* were extracted using TRIzol Reagent (Invitrogen) separately, then treated with DNase I (TaKaRa). RNA quality was tested by 1% agarose gel electrophoresis and the concentration determined using Nanodrop spectrophotometer (Thermo). RNA pools were prepared by mixing equal amounts of the three biological replicates for each age rhizome. Transcriptome data of 1-, 2- and 3-year old rhizomes were acquired using based on the Illumina hiseq Xten PE150 platform, by Novogene Co. (Beijing, China). The raw paired end reads were trimmed and quality controlled by SeqPrep (https://github.com/jstjohn/SeqPrep) and Sickle (https://github.com/najoshi/sickle) with default parameters. The sequencing data with high-quality reads were gathered using fastp (version 0.19.5, https://github.com/OpenGene/fastp). In short, adapter’s contamination, bases with low quality, reads having (≥ 10%) ambiguous bases, and reads having ˂20 bp were removed. Then clean data from all three age rhizomes were used to do de novo assembly with Trinity (version 2.8.5, http://trinityrnaseq.sourceforge.net/). The datasets generated and analyzed during the current study are available in the Sequence Read Archive (SRA) repository (https://www.ncbi.nlm.nih.gov/sra/PRJNA698794). The transcriptome data have been uploaded to SRA (BioProject ID PRJNA698794, https://www.ncbi.nlm.nih.gov/sra/PRJNA698794).

Sequence annotation and classification were referenced to the method of Ramya et al. [22]. For functional annotation, all the assembled transcripts were searched against the Nr (NCBI protein non-redundant), COG (Clusters of Orthologous Groups of proteins) and KEGG (Kyoto Encyclopedia of Genes and Genomes) databases using BLASTx to identify the proteins that had the highest sequence similarity with the given transcripts to retrieve their function annotations and a typical cut-off E-values less than 1.0 × 10^–5^ was set. BLAST2GO (http://www.blast2go.com/b2ghome) program was used to get GO (Gene Ontology) annotations of unique assembled transcripts. Metabolic pathway analysis was performed using the KEGG (http://www.genome.jp/kegg/). For other sequences not involved in the BLAST searches, we used the TransDecoder program (https://github.com/TransDecoder/TransDecoder) to predict coding sequence (CDS) and orientation.

### Analysis of differentially expressed genes

Differential expression of unigenes among three age rhizomes of *A. chinensis* were determined using DESeq2 (Version 1.24.0) software. DESeq2 provides statistical routines for determining differential expression in digital gene expression data using a model based on the negative binomial distribution. For functional-enrichment analysis including GO and KEGG were performed to identify which DEGs were significantly enriched in GO terms and KEGG metabolic pathways at Bonferroni-corrected P-adjust < 0.05 compared with the whole-transcriptome background. GO functional enrichment and KEGG pathway analysis were carried out by Goatools (version 0.6.5, https://github.com/tanghaibao/Goatools) and KOBAS (http://kobas.cbi.pku.edu.cn/kobas3/?t=1).

Differences in gene expression were evaluated using the chi-square test and the false discovery rate (FDR) was controlled. The FPKM (fragments per kilobase of exons model per million mapped and reads) were assigned to normalize reads expression values. This study took FDR value of (≤ 0.05) and (log_2_FC ≥ 1) as a criterion for screening DEGs. Corrected P-adjust < 0.05 were used as thresholds for “enriched” DEGs. Heat maps were generated to display genes with significantly altered expression at the three ages.

### Quantitative real-time PCR

To confirm the reliability of the RNA sequencing analysis, qRT-PCR analyses were performed using samples from the same 1-, 2- and 3-year old rhizomes as used for RNA-seq. Eleven genes (cluster-15114.3, cluster-8388.71372, cluster-8388.203329, cluster-388.168445, cluster-8388.64828, cluster-8388.299573, cluster-8388.162261, cluster-8388.157231, cluster-8388.172353, cluster-8388.295361 and cluster-8388.295722), with key functions in sesquiterpenoid biosynthetic pathway, as well as in pigment, amino acid, hormone, and transcription factor functions, were randomly selected for qRT-PCR analysis. Primers for qRT-PCR were designed using Primer v5.0 and synthesized by Sangon Biotech (Shanghai, China) Co., Ltd. cDNAs were reverse-transcribed from total RNA using the PrimeScript RT reagent Kit (TaKaRa), and qRT-PCR analyses were performed on an BIO-RAD CFX Connect™ Real-Time PCR detect System (Singapore Biosystems). Relative expression data were normalized to those of the *UBQ2* gene, which was used as an internal control [36]. Each qRT-PCR experiment was repeated three times. The relative expression of each gene was calculated using the 2^−∆∆Ct^ method [19] and the SD was calculated among three biological replicates. All primers used are listed in Supplementary Table S1.

Validation of the seven DEGs related to sesquiterpenoid and triterpenoid biosynthesis using qRT-PCR according to method of reliability confirmation for the RNA sequencing. The primers are listed in Supplementary Table S2.

## Results

### The atrctylodin content in 1-, 2- and 3-year old *A. chinensis* rhizomes

As atrctylodin is the main and index bioactive constituent in *A. chinensis*, its levels in rhizome from 1-, 2- and 3-year old *A. chinensis* plants were measured by HPLC analysis, with atrctylodine contents (%) recorded as 0.2252, 0.2378 and 0.2939, respectively (Table 1, Supplementary Fig. S1). The atractylodin content in 3-year old rhizome was significantly higher than 1- and 2-year old rhizomes (Table 1). These data showed that cultivation year had marked effect on atrctylodin content of *A. chinensis* rhizome, however, the molecular mechanisms underlying the higher atrctylodin content in 3-year old rhizome is unclear.

**Table 1:** Atractylodin content (%) in rhizomes of 1-, 2- and 3-year old *A. chinensis*

Cultivation year	Content (%)
1	0.2252
2	0.2378
3	0.2939*

Note: Asterisks shows significant differences based on the *t-test* (*p < 0.05; **p < 0.01). The atractylodin content of 1-year old rhizome was considered as control. The values are representative of three biological replicates

### Sequencing analysis and de novo assembly

To study the molecular mechanisms involved in the relationship between increased atrctylodin content and *A. chinensis* cultivation year, transcriptome sequencing was conducted. After filtering out adapter sequences and reads ≤ 50 bp, 58,394,019, 51,583,471, and 59,107,505 high-quality (HQ) reads were obtained from 1-, 2- and 3-year old *A. chinensis* rhizomes, respectively. Reads from the three samples were also pooled and the above steps repeated, resulting in identification of 143,616 unigenes (mean length 825 bp, N50 length 1121 bp). The GC content of the reads and unigenes was in the range 45.18% ~ 45.56% (Table 2). Analysis of length distribution demonstrated that 16% unigenes were > 1 kb.

**Table 2:** Summary of Illumina sequencing and assembly of *A. chinensis*

	1- year-old rhizome	2- year-old rhizome	3- year-old rhizome
Number of raw reads	58,573,338	51,757,902	59,322,720
Number of clean reads	58,394,019	51,583,471	59,107,505
Q30 (%)^a^	92.61	92.24	92.64
GC content (%)	45.53	45.18	45.56
Number of unigene^a^		143, 616	
Length of unigene (bp) ^a^		343,909,490	
Average length of unigene (bp) ^a^		825	
N50 of unigene (bp) ^a^		1121	

^a^ The total number of contigs and singletons

### Functional annotation and classification

A total of 56,759 unigenes (39.52% of the total assembled unigenes) had matches in the Nr database, with 37,475 (26.09%), 31,272 (21.77%), 6,540 (4.55%), 39,372 (27.41%) and 31,424 (14.92%) unigenes showing significant similarity to sequences in the Swiss-Prot, Pfam, COG, GO and KEGG databases, respectively (Table 3).

**Table 3:** Summary of annotations of the unigenes in the *A. chinensis* rhizomes transcriptome against public databases

Database	Unigenes	Percentage (%)
Nr	56,759	39.52
Swiss-Prot	37,475	26.09
Pfam	31,272	21.77
COG	6,540	4.55
GO	39,372	27.41
KEGG	31,424	14.92
Total annotation	58,466	40.71

Note: the link of databases: Nr (version 2019.6.26; http://ftp.ncbi.nlm.nih.gov/blast/db/), Swiss-Prot (version 2019.7.1; http://web.expasy.org/docs/swiss-prot_guideline.html), Pfam (version 32.0; http://pfam.xfam.org/), COG (eggnog version 5.0; http://www.ncbi.nlm.nih.gov/COG/), GO (http://www.geneontology.org/), KEGG (version 2017.08; http://www.genome.jp/kegg/)

**Fig. 1:**
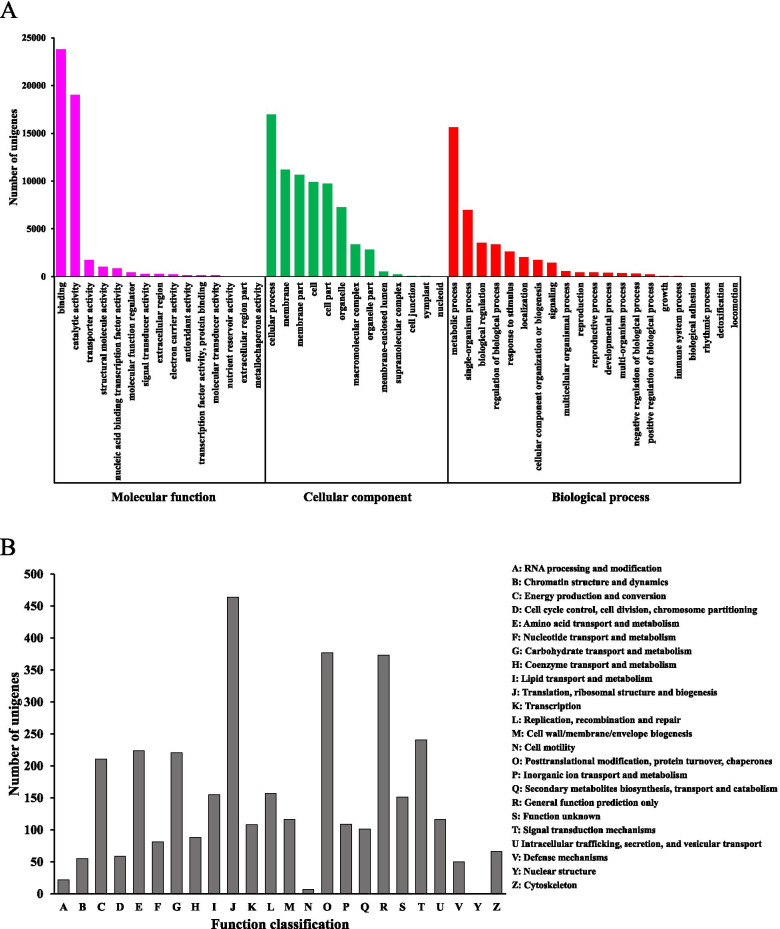
GO and COG classification of assembled unigenes. (**A**) GO classification. The assembled unigenes were classified into three main categories in GO classification. The *y*-axis indicates the number of unigenes in category, and *x*-axis indicates the GO classification. (**B**) COG classification. The assembled unigenes were classified into 24 categories in COG classification. The *y*-axis indicates the number of unigenes in category, and *x*-axis indicates the COG classification

The counts of the unigenes in the three GO categories included biological process (57,008 unigenes), cellular component (55,906 unigenes) and molecular function (47,730 unigenes) with 47 sub categories (Fig. 1A). In total, 6,540 unigenes were annotated and grouped into 24 COG classifications (Fig. 1B), among which, the cluster for “translation, ribosomal structure and biogenesis” (464 unigenes, 7.10% of the total COG annotated unigenes) accounted for the largest proportion, followed by “posttranslational modification, protein turnover, chaperones” (377 unigenes, 5.80%) and “general function prediction only” (373 unigenes, 5.70%).

**Fig. 2:**
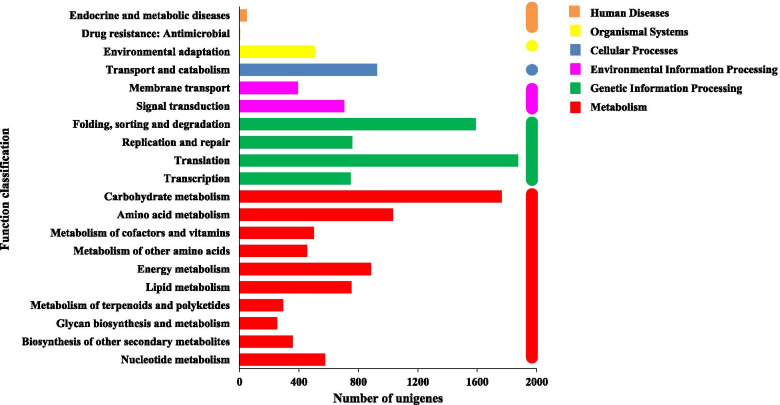
Functional classification and pathway assignment of assembled unigenes by KEGG. The assembled unigenes were classified into six main categories in KEGG classification. The *x*-axis indicates the number of unigenes in category, and *y*-axis indicates the KEGG classification

KEGG pathway analysis was performed to functionally classify biochemical pathways active in 1-, 2- and 3-year old *A. chinensis* rhizomes. A total of 31,424 unigenes were assigned to 6 KEGG categories with 130 sub categories: “metabolism”, “genetic information processing”, “environmental information processing”, “cellular processes”, “organismal systems” and “Human diseases” (Fig. 2).

Of KEGG secondary metabolic pathways, most unigenes were assigned to “phenylpropanoid biosynthesis” (723 unigenes, ko00940), “terpenoid backbone biosynthesis” (517 unigenes, ko00900), “carotenoid biosynthesis” (394 unigenes, ko00906), and “sesquiterpenoid and triterpenoid biosynthesis” (204 unigenes, ko00909) (Table 4). A total of 240 unigenes were identified as key enzyme genes in the MVA pathway, with 169 unigenes in the MEP pathway, and 131 unigenes in the sesquiterpenoid and triterpenoid biosynthetic pathway (Table 5). The discovery of these genes related to sesquiterpenoid and triterpenoid biosynthetic pathway may help us to elucidate the molecular mechanisms underlying the higher atrctylodin content in 3-year old rhizomes.

**Table 4:** Number of unigenes related to secondary metabolites in *A. chinensis*

Secondary metabolites biosynthetic pathway	Number of unigenes	Pathway ID
Phenylpropanoid biosynthesis	723	ko00940
Terpenoid backbone biosynthesis	517	ko00900
Carotenoid biosynthesis	394	ko00906
Sesquiterpenoid and triterpenoid biosynthesis	204	ko00909
Tropane, piperidine and pyridine alkaloid biosynthesis	194	ko00960
Zeatin biosynthesis	170	ko00908
Monobactam biosynthesis	157	ko00261
Isoquinoline alkaloid biosynthesis	138	ko00950
Stilbenoid, diarylheptanoid and gingerol biosynthesis	116	ko00945
Limonene and pinene degradation	116	ko00903
Flavonoid biosynthesis	82	ko00941
Diterpenoid biosynthesis	71	ko00904
Monoterpenoid biosynthesis	69	ko00902
Caffeine metabolism	55	ko00232
Brassinosteroid biosynthesis	44	ko00905
Flavone and flavonol biosynthesis	38	ko00944
Glucosinolate biosynthesis	28	ko00966
Betalain biosynthesis	20	ko00965
Anthocyanin biosynthesis	8	ko00942
Isoflavonoid biosynthesis	4	ko00943
Indole alkaloid biosynthesis	3	ko00901

**Table 5:** Discovery of unigenes involved in sesquiterpenoid and triterpenoid biosynthesis in *A. chinensis*

Pathway	Enzymes name	Abbreviation	Number of unigenes
MVA	Acetyl-CoA C-acetyltransferase	AACT	27
	3-hydroxy-3-methylglutaryl coenzyme A synthase	HMGS	35
	3-hydroxy-3-methylglutaryl coenzyme A reductase	HMGR	31
	mevalonate kinase	MK	2
	phosphomevalonate kinase	PMK	38
	Mevalonate-5-pyrophosphate decarboxylase	MDC	8
	Geranyl diphosphate synthase	GPPS	39
	Farnesyl diphosphate synthase	FPPS	10
	Isopentenyl-diphosphate delta-isomerase	IPPI	19
	Mevalonate pyrophosphate decarboxylase	MVD	8
	Isopentenyl diphosphate isomerase	IDI	13
	Farnesyl diphosphate synthase	FDPS	10
MEP	1-deoxy-*D*-xylulose-5-phosphate synthase	DXPS	74
	1-deoxy-*D*-xylulose-5-phosphate reductoisomerase	DXR	22
	2-*C*-methyl-*D*-erythritol 4-phosphate cytidylyl transferase	MCT	22
	4-diphosphocytidyl-2-*C*-methyl-*D*-erythritol kinase	CMK	1
	4-hydroxy-3-methyl but-2-(*E*)-enyl-diphosphate synthase	HDS	36
	4-hydroxy-3-methyl but-2-(*E*)-enyl-diphosphate reductase	HDR	14
Sesquiterpenoid and triterpenoid biosynthesis	Beta-caryophyllene synthase	QHS1	5
	Germacrene D synthase	GDS	9
	Germacrene A synthase	GAS	1
	Sesquiterpene synthase	TPS	7
	Squalene synthase	SS	36
	Squalene epoxidase	SE	71
	Dammarenediol-II synthase	DS	2

### Differential expression of transcripts in *A. chinensis* rhizomes from different cultivation year

**Fig. 3:**
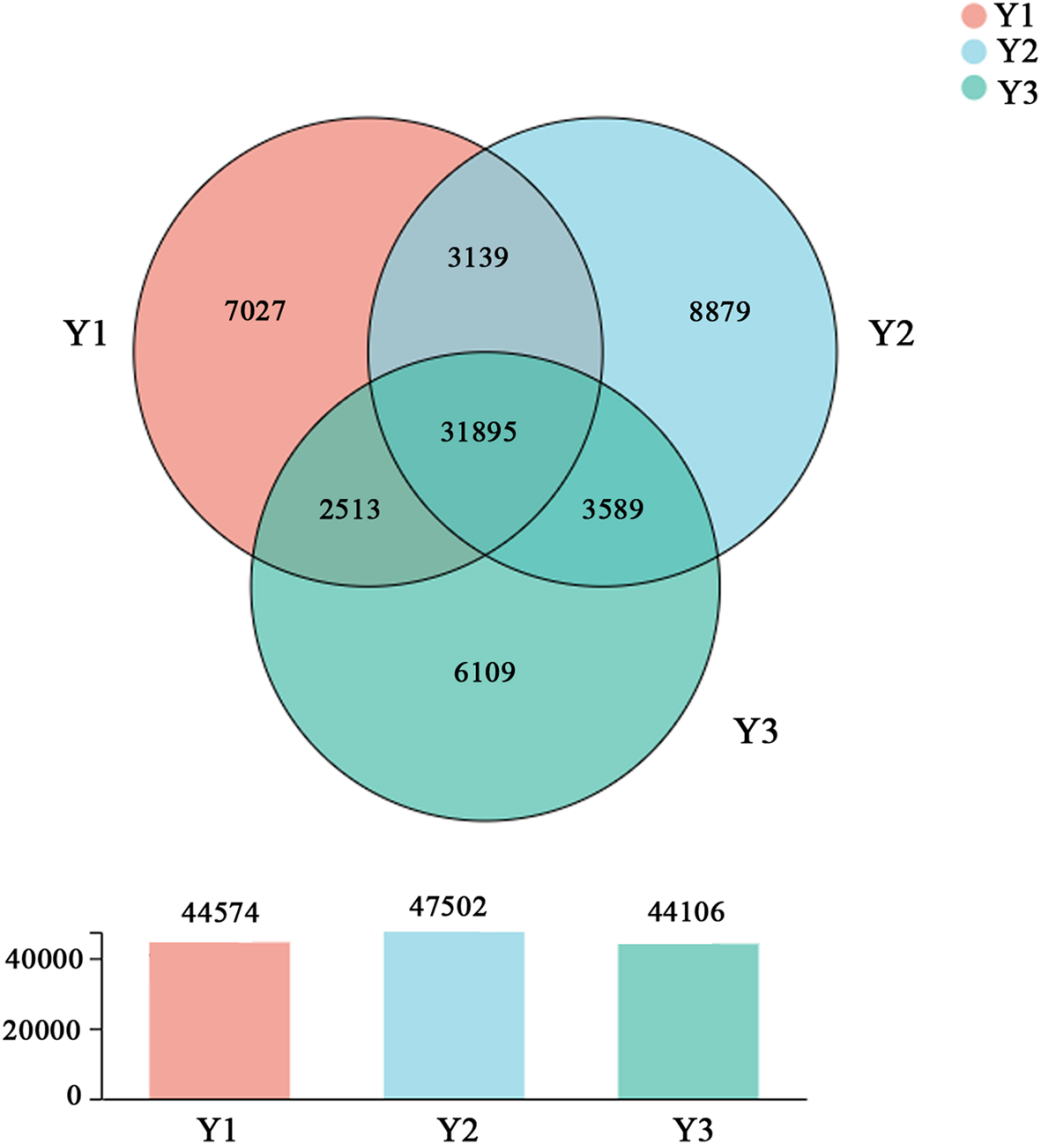
Venn diagram of unigenes from 1-, 2- and 3-year old rhizomes of *A.chinensis*. The diagram above shows the overlapping and specific unigenes in 1-, 2- and 3-year old rhizomes. The column chart below shows the total number unigenes in 1-, 2- and 3-year old rhizomes, respectively

To compare the unigenes from different age *A. chinensis* rhizomes, a Venn diagram was constructed (Fig. 3). The results showed that 31,895 unigenes were shared by all three age rhizomes. A total of 7,027, 8,879 and 6,109 unigenes were specific to 1-, 2- and 3-year old *A. chinensis* rhizomes, respectively, with the 2-year-old *A. chinensis* rhizome having the highest number of unique unigenes.

**Fig. 4:**
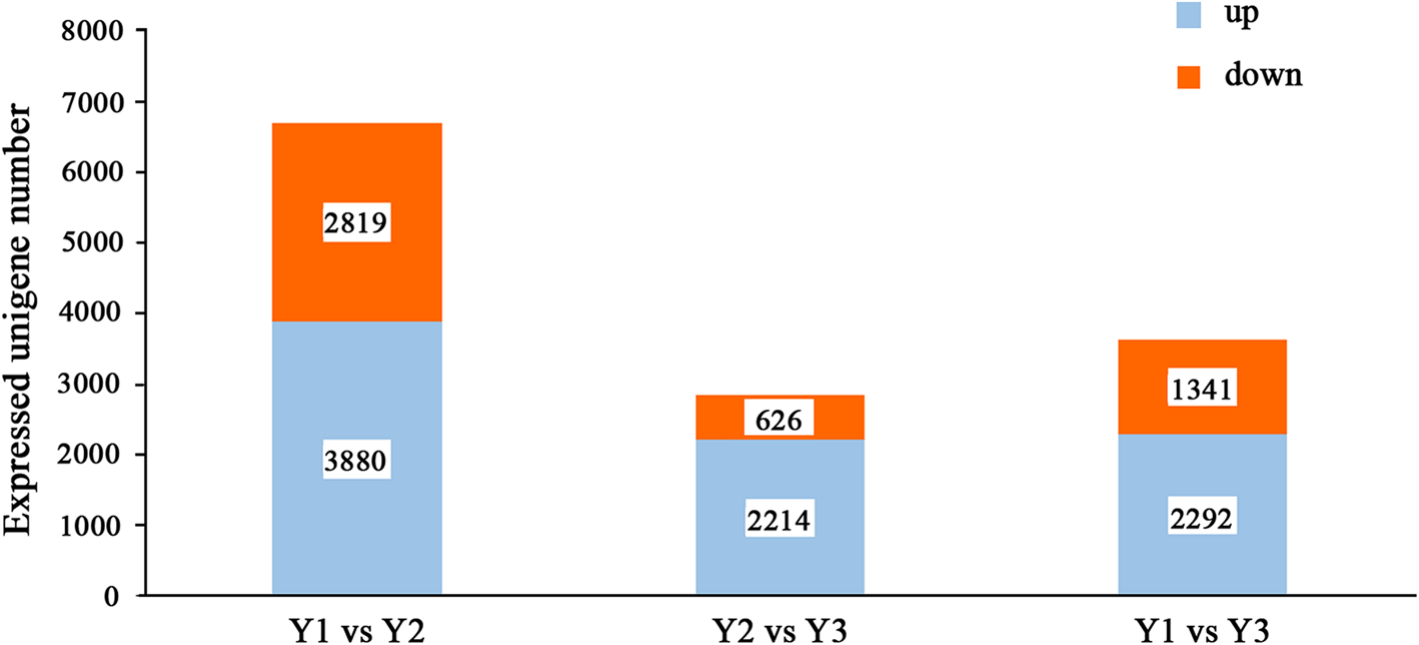
The number of up-down regulated DEGs of Y1 vs Y2, Y1vs Y3 and Y2 vs Y3. The up regulated DEGs are marked with red, the down regulated DEGs are marked with blue

To identify DEGs among the three age rhizomes, the tag frequencies of 1- vs 2-year-old (Y1 vs Y2) rhizome, 2- vs 3-year old (Y2 vs Y3) rhizome and 1- vs 3-year old (Y1 vs Y3) rhizomes were assessed, with 6,699, 2,840, and 3,633 DEGs detected between the three pair comparisons, respectively (Fig. 4). Y1 vs Y2, Y2 vs Y3 and Y1 vs Y3, revealed 3,880, 2,214 and 2,292 up-regulated genes. There were more up-regulated than down-regulated genes in Y1 vs Y2, with the opposite detected in the Y2 vs Y3 and Y1 vs Y3 comparisons.

**Fig. 5:**
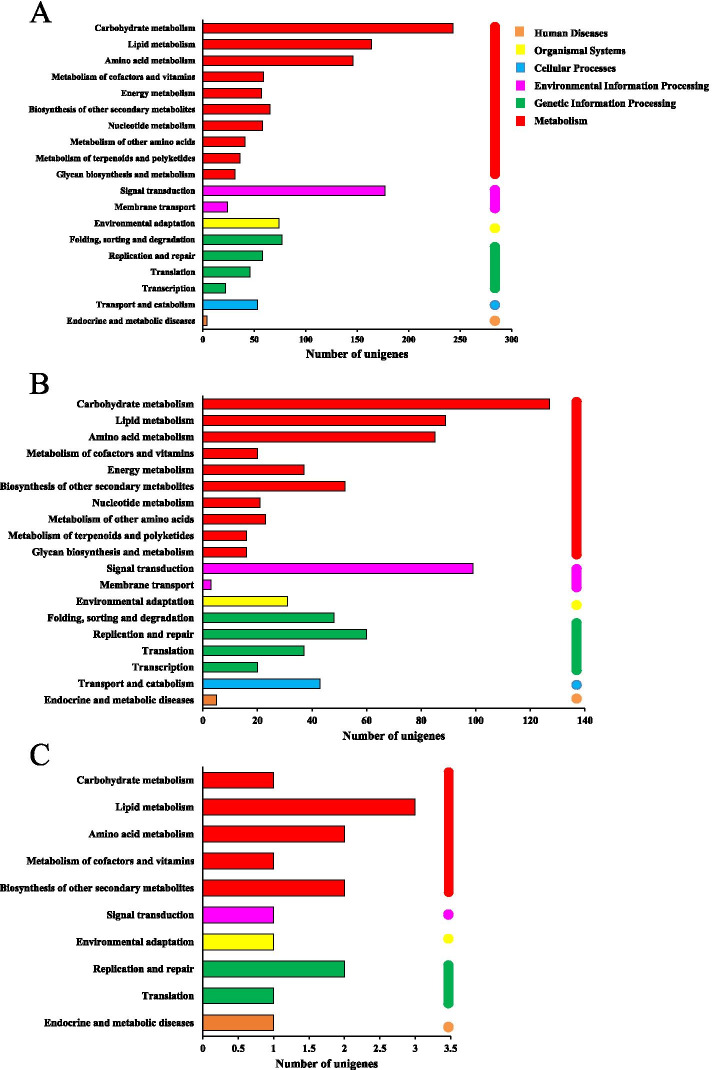
The functional classification of DEGs in KEGG pathways. (**A**) Y1 vs Y2; (**B**) Y1 vs Y3; (**C**) Y2 vs Y3. The assembled unigenes were classified into six main categories in KEGG classification. The *x*-axis indicates the number of unigenes in category, and *y*-axis indicates the KEGG classification

KEGG pathway enrichment analysis of all DEGs was performed to characterize the complex biological behaviors. The enriched pathways are presented in Fig. 5, and reflected the preferential biological functions of samples from different age rhizomes. Hierarchical clustering of all DEGs indicated that overall unigenes enrichment characteristics were similar between the Y1 vs Y2 and Y1 vs Y3 comparisons (Fig. 5A and 5B), with genes involved in “Carbohydrate metabolism”, “Signal transduction”, “Amino acid metabolism”, “Lipid metabolism” and “Biosynthesis of secondary metabolites” over-expressed. In Y2 vs Y3, genes involved in “Lipid metabolism”, “Amino acid metabolism”, “Biosynthesis of secondary metabolites” and “Replication and repair” were overexpressed (Fig. 5C).

**Fig. 6:**
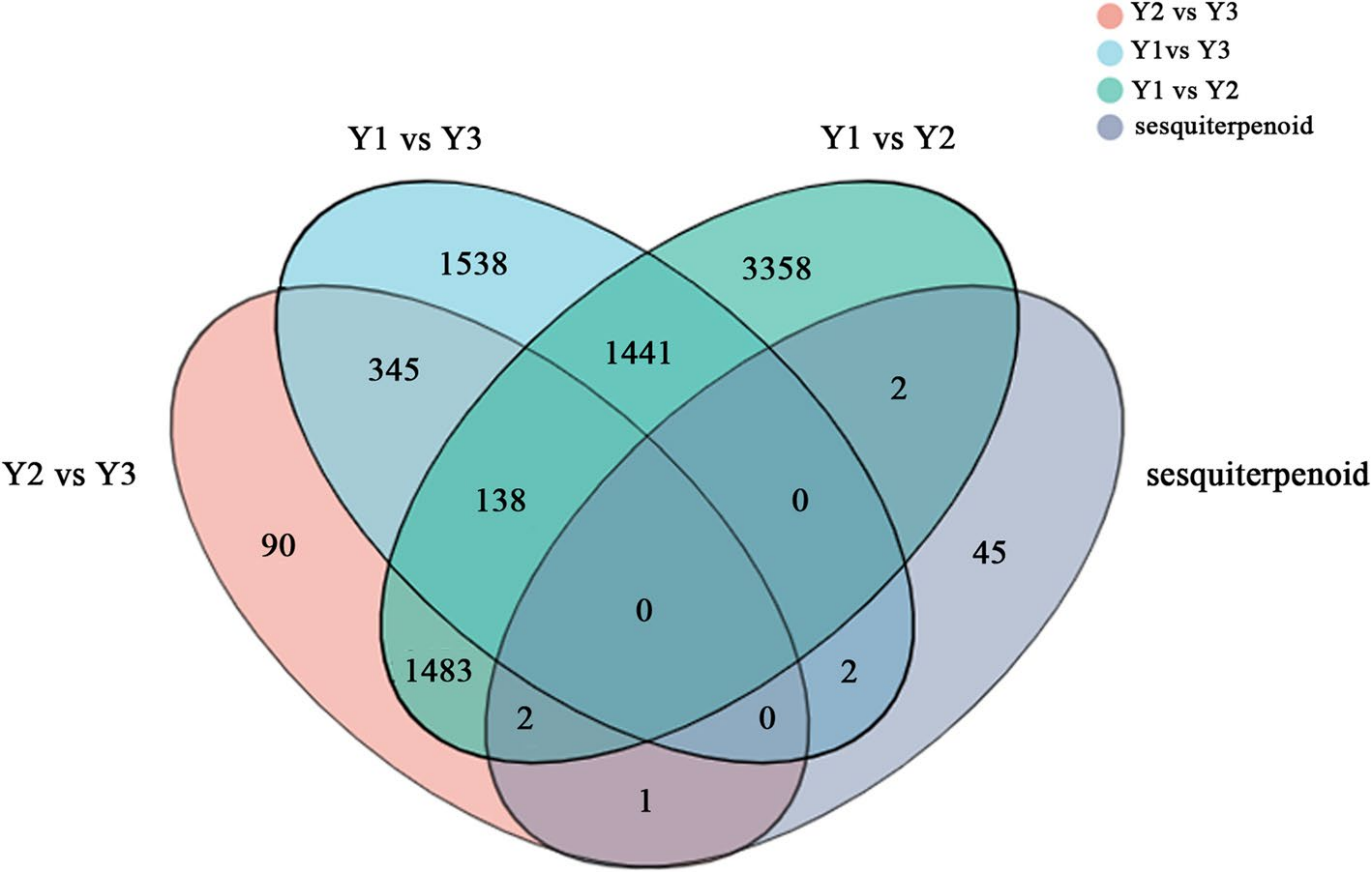
A venn diagram of DEG statistics from Y1 vs Y2, Y1 vs Y3, Y2 vs Y3 and sesquiterpenoid genes. The diagram shows the overlapping unigenes in Y1 vs Y2, Y1 vs Y3, Y2 vs Y3 and sesquiterpenoid. The results reveals 7 DEGs in sesquiterpenoid biosynthesis pathway

Pathways involved in bioactive compounds metabolism are of particular interest in medicinal plants. DEGs involved in “Biosynthesis of secondary metabolites” were overexpressed in all three pair comparisons; 52 genes related to sesquiterpenoid and triterpenoid biosynthesis were detected, of which seven were differentially expressed in Y1 vs Y2, Y1 vs Y3 and Y2 vs Y3 (Fig. 6).

**Fig. 7:**
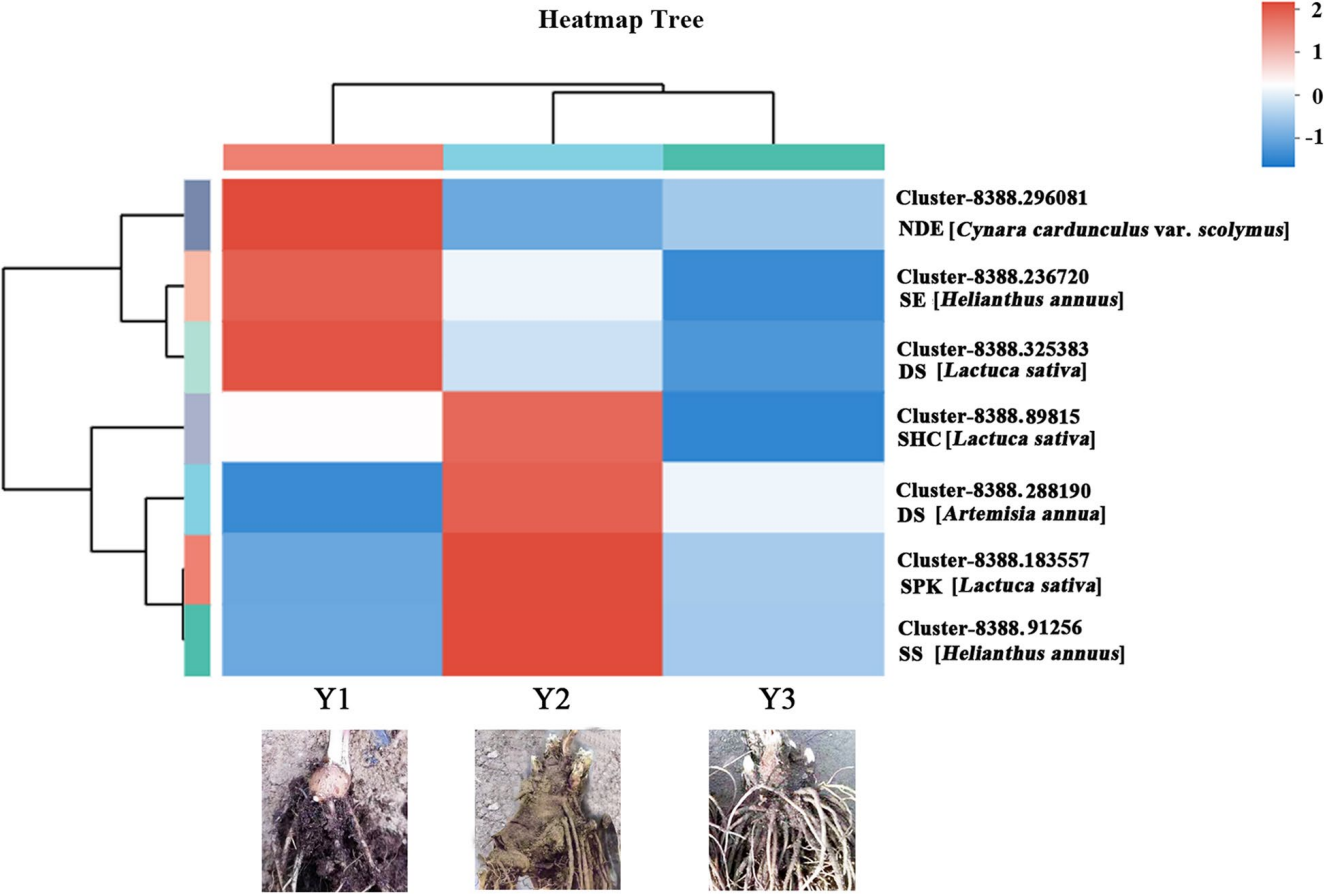
Heatmap of expressions for DEGs related to sesquiterpenoid and triterpenoid biosynthetic pathway. Heatmap shows the expression patterns of seven DEGs genes in the 1-, 2- and 3-year old rhizomes. The red color indicates higher expression while blue indicated lower expression

**Fig. 8:**
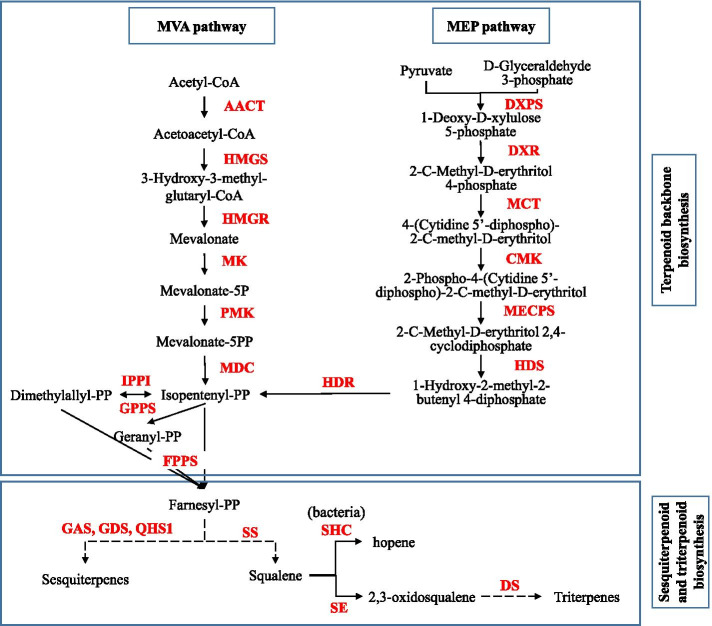
Flow diagram of putative sesquiterpenoid and triterpenoid biosynthetic pathway. The red letters represent key enzymes for the action of sesquiterpenoid and triterpenoid biosynthetic pathway. Solid line represented directly catalytic reaction, and dotted line for indirectly catalytic reaction

Heatmap trees were constructed based on gene expression levels, to further investigate the seven differentially expressed genes involved in sesquiterpenoid and triterpenoid biosynthetic pathway, including NAD-dependent epimerase/dehydratase (NDE), squalene/phytoene synthase (SS), squalene-hopene cyclases (SHC), squalene epoxidase (SE), dammarenediol-II synthase (DS), and serine/threonine-protein kinase SRK2E (SPK). All of these seven DEGs down-regulated expression in 3-year old rhizome, comparing with 1- and 2-year old rhizomes (Fig. 7). Notably, five of these DEGs encoded four key enzymes: SS, SHC, SE and DS. These four enzymes directly related to squalene biosynthesis and subsequent catalytic action. According to putative pathway, squalene is the first precursor in triterpenoid biosynthetic pathway (Fig. 8).

### Validation of RNA-seq analysis by qRT-PCR

**Fig. 9:**
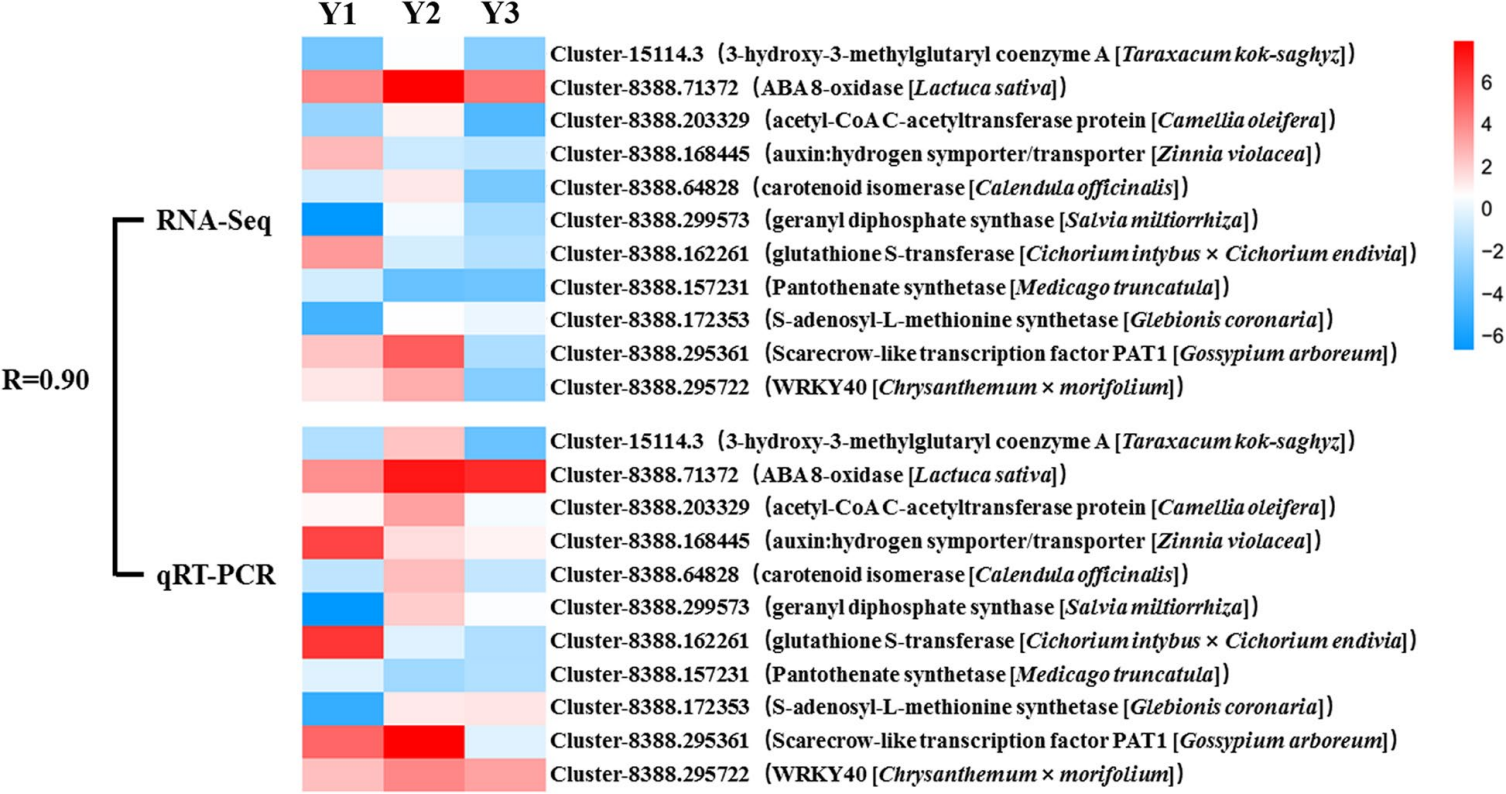
QRT-PCR validation of transcriptome sequencing analysis*.* Heat map showed the mean value of transcript levels detected in three biological replicates. Relative transcript levels as detected by RNA-Seq (top) or by qRT-PCR (bottom) were shown by color scales. R, correlation coefficient value between RNA-seq data and qRT-PCR data. The red color indicates higher expression while blue indicated lower expression

To confirm the reliability of the RNA sequencing analysis, eleven genes representing key genes in sesquiterpenoid and triterpenoid biosynthetic pathway, as well as in pigment, amino acid, hormone and transcription factor functions, were selected for qRT-PCR analysis. The result demonstrated similar expression patterns to those determined by RNA sequencing, with a Pearson*’*s correlation co-efficient between qRT-PCR and RNA-seq data of 0.9 (Fig. 9).

**Fig. 10:**
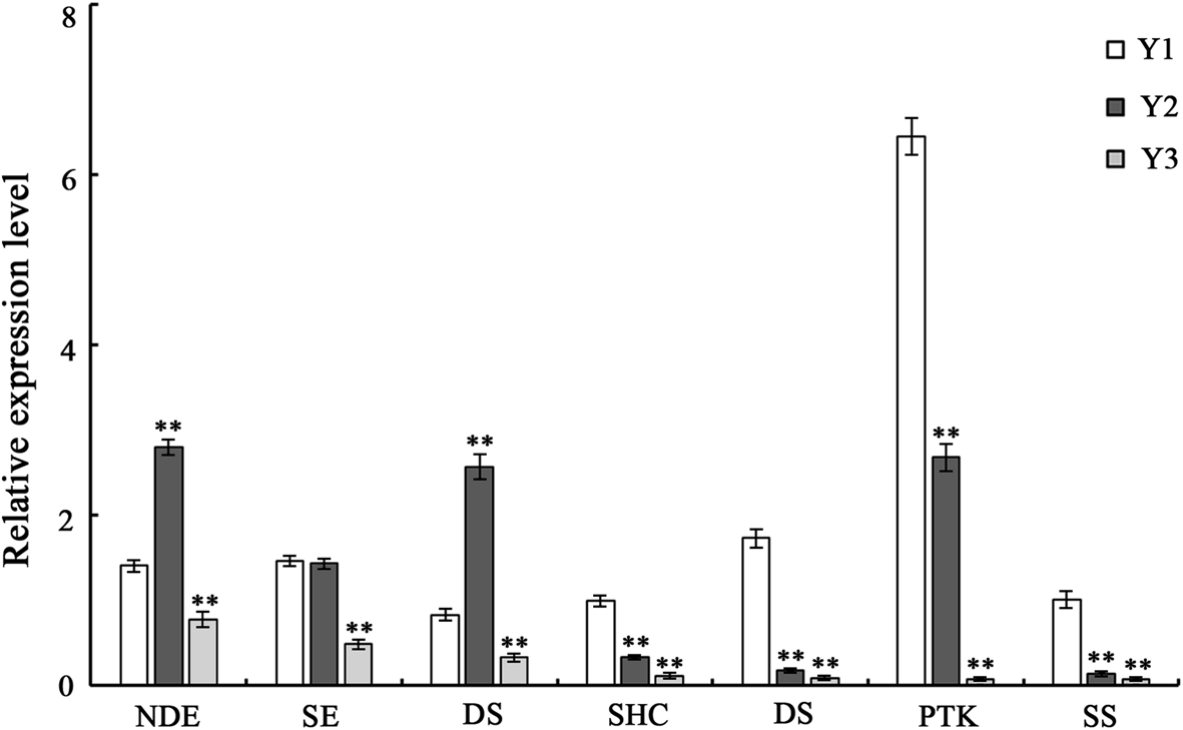
QRT-PCR analysis of seven DEGs involved in sesquiterpenoid and triterpenoid biosynthetic pathway. QRT-PCR was used to validate the relative expression levels of seven selected genes revealed by RNA-seq. The relative expression levels of 1-year old rhizome were considered as controls. The values are representative of three biological replicates. *Error bars* indicate standard errors of the mean. Asterisks indicate significant differences among 1-, 2- and 3-year old rhizomes based on the *t-test* (*p < 0.05; **p < 0.01). NDE: Cluster-8388.296081; SE: Cluster-8388.236720; DS: Cluster-8388.325383; SHC: Cluster-8388.89815; DS: Cluster-8388.288190; SPK: Cluster-8388.183557; SS: Cluster-8388.91256

Further validation of seven DEGs related to sesquiterpenoid and triterpenoid biosynthetic pathway was performed by qRT-PCR. The relative expression levels of these seven DEGs noted in 3-year old rhizome were significantly lower than those in 1- and 2-year old rhizomes (Fig. 10). These results are consistent with the data of transcriptomic sequencing analysis.

## Discussion

As genome data for the *Atractylodes* genus are not yet available, Illumina-based RNA sequencing was performed to characterize the *A. chinensis* transcriptome. We obtained 143, 616 unigenes, of which 40.71% could be functionally annotated based on public databases. In addition, the qRT-PCR results demonstrated similar expression patterns of eleven randomly selected genes to those determined by RNA-seq analysis, demonstrating the reliability of our *A. chinensis* transcriptome data.

Transcriptomic analysis to investigate sesquiterpenoid accumulation patterns in different tissues (leaf and rhizome) of *A. lancea* discovered 69 unigenes in the MVA pathway, including nine key enzymes, and 28 unigenes in the MEP pathway, involving seven key enzymes [3]. In this study, we investigated the sesquiterpenoid accumulation patterns in *A. chinensis* of different cultivation year and discovered 240 unigenes in the MVA pathway, involving twelve key enzymes, and 169 unigenes in the MEP pathway, involving six crucial enzymes. It inferred more differences between different age rhizomes than different tissues from the same individual. These data will facilitate further study of the molecular mechanisms underlying sesquiterpenoid accumulation.

In this study, we found that atractylodin content in *A. chinensis* rhizome increased with the increase of cultivation year. Li et al. [17] confirmed that the age of cultivation medicinal plants was important in increasing saponins production in *Panax notoginseng* rhizomes. Based on this natural phenomena, we performed differential expression analysis using transcriptome data from 1-, 2- and 3-year old rhizomes, to identify candidate DEGs encoding key enzymes in sesquiterpenoid and triterpenoid biosynthetic pathway. Differential gene expression patterns were further investigated to profile global gene expression differences between Y1 vs Y2, Y2 vs Y3 and Y1 vs Y3. Most DEGs between Y1 vs Y2 and Y1 vs Y3 were assigned to 19 metabolic pathways, including signal transduction, primary metabolic pathways (carbohydrate metabolism, amino acid metabolism and lipid metabolism), and biosynthesis of other secondary metabolites. In Y2 vs Y3, DEGs were assigned to 10 metabolic pathways, of which lipid metabolism, amino acid metabolism, replication and repair, and biosynthesis of other secondary metabolites comprised a higher percentage. These data indicate that the metabolic characteristics of 2-year old rhizome are more similar to those of 3-year old rhizome, relative to 1-year old rhizome. Further, the metabolic characteristics of DEGs were consistent with the rhizome’s physiological function as a storage organ for photosynthetic products and bioactive compounds. These data demonstrated that vitality of medicinal plants and the production of secondary metabolic became increased over the cultivation year, likely because they are crucial for defense against stress in older plants.

Further analysis of DEGs provided information crucial for investigation of the molecular mechanisms involved in sesquiterpenoid biosynthesis and accumulation in *A. chinensis*. Seven key genes related to sesquiterpenoid and triterpenoid biosynthesis were discovered by analysis DEGs between Y1 vs Y2, Y1 vs Y3, and Y2 vs Y3. Of the seven key genes, five encoding four enzymes: SE, SHC, SS and DS. The biological production of sesquiterpenoid and triterpenoid is an extremely complicated process, with synthesis occurring via MEP and MVA pathway. Many enzymes are involved in the process of isoprenenyl diphosphate (IPP) biosynthesis catalysis, which was then catalyzed toward two biosynthesis branch, sesquiterpenoids biosynthesis branch and squalene biosynthesis branch.

The identified four enzymes, SE, SHC, SS and DS, play important role in squalene biosynthesis and the subsequent catalytic reactions in this biosynthesis branch. The enzyme SS as a key enzyme in the terpenoid biosynthetic pathway catalyzes the synthesis of the first precursor of terpenoid compounds, squalene [7, 15, 23, 35, 39]. The SHC enzyme can catalyze the formation of hopene from its precursor squalene [21, 25], toward triterpenoid or steroid biosynthesis. The enzyme, SE, which catalyzes the oxidation of squalene to 2, 3-oxysqualene, is a rate-limiting enzyme in the sterol biosynthesis [31]. DS enzyme is the first dedicated enzyme for ginsenoside biosynthesis, one of triterpenoid compounds [28].

The enzymes SS and SE has been found the rate-limiting enzymes involved in sterol and cholesterol biosynthesis [7, 26]. The substrate for SS is farnesyl diphosphate (FPP), which was the important substrate of sesquiterpenoid and triterpenoid biosynthesis. In the case of suppression of enzyme SS activity was observed induction of sesquiterpenoid cyclase, toward the sesquiterpenoid biosynthetic pathway [40]. Evidence suggested that inhibition of either SS or SE was found to trigger a severalfold increase in enzyme activity of HMGR [31]. HMGR is the first rate-controlling enzyme for the synthesis of variety of isoprenoids via MVA pathway [8, 10, 30]. This study revealed that the four enzymes SS, SHC, SE and DS down-regulated expression in 3-year old rhizome. This data corresponded to the higher content of sesquiterpenoid in 3-year old rhizome, and confirmed by qRT-PCR. We would like to infer that the sesquiterpenoid biosynthesis branch is the main biosynthetic pathway in 3-year old rhizome of *A. chinensis*. This study reported the results of comparative transcriptome analysis and identified key enzyme genes, laid a solid foundation for investigation of production sesquiterpenoid in *A. chinensis*.

## Supplementary Information

**Additional file 1: Table S1.** Primers of qRT-PCR for validation of the reliability of RNA-seq analysis. **Table S2.** Primers of qRT-PCR for validation of the seven DEGs involved in sesquiterpenoid and triterpenoid biosynthetic pathway.

**Additional file 2: Fig. S1** The content of atractylodin in A. chinensis rhizomes. A. standard (20 mg∙ml^−1^); B. 1-year old rhizome; C. 2-year old rhizome; D. 3-year old rhizome.

## Data Availability

The datasets analyzed during the current study are available in the SRA (BioProject ID PRJNA698794, https://www.ncbi.nlm.nih.gov/sra/PRJNA698794) repository. The public access to the databases is open.

## Abbreviations

*A. **chinensis*: *Atractylodes* *chinensis* (DC.) Koids.
DEGs: Differentially expressed genes
qRT-PCR: Quantitative real-time PCR
MVA: Mevalonate
MEP: Methylerythritol phosphate
AACT: Acetyl-CoA C-acetyltransferase
HMGS: 3-Hydroxy-3-methylglutaryl coenzyme A synthase
HMGR: 3-Hydroxy-3-methylglutaryl coenzyme A reductase
MK: Mevalonate kinase
PMK: Phosphomevalonate kinase
MDC: Mevalonate-5-pyrophosphate decarboxylase
GPPS: Geranyl diphosphate synthase
FPPS: Farnesyl diphosphate synthase
IPPI: Isopentenyl-diphosphate delta-isomerase
MVD: Mevalonate pyrophosphate decarboxylase
IDI: Isopentenyl diphosphate isomerase
FDPS: Farnesyl diphosphate synthase
DXPS: 1-Deoxy-*D*-xylulose-5-phosphate synthase
DXR: 1-deoxy-*D*-xylulose-5-phosphate reductoisomerase
MCT: 2-*C*-Methyl-*D*-erythritol 4-phosphate cytidylyl transferase
CMK: 4-Diphosphocytidyl-2-*C*-methyl-*D*-erythritol kinase
HDS: 4-Hydroxy-3-methyl but-2-(*E*)-enyl-diphosphate synthase
HDR: 4-Hydroxy-3-methyl but-2-(*E*)-enyl-diphosphate reductase
QHS1: Beta-caryophyllene synthase
GDS: Germacrene D synthase
GAS: Germacrene A synthase
TPS: Sesquiterpene synthase
SS: Squalene synthase
SE: Squalene epoxidase
DS: Dammarenediol-II synthase
IPP: Isoprenenyl diphosphate
FPP: Farnesyl diphosphate
