# Supplementary material for: Comparative transcriptome analysis reveals sesquiterpenoid biosynthesis among 1-, 2- and 3-year old Atractylodes chinensis
Source: BMC Plant Biol. 2021 Jul 27;21:354. doi: 10.1186/s12870-021-03131-1 (PMC8314494; doi:10.1186/s12870-021-03131-1)
Supplement: Supplementary file 1 — Additional file 1: Table S1. Primers of qRT-PCR for validation of the reliability of RNA-seq analysis. Table S2. Primers of qRT-PCR for validation of the seven DEGs involved in sesquiterpenoid and triterpenoid biosynthetic pathway. [file 12870_2021_3131_MOESM1_ESM.docx]

Supplementary Table S1 Primers of qRT-PCR for validation of the reliability of RNA-seq analysis

| clusters | primers (5’→3′) |  |
| --- | --- | --- |
| cluster-15114.3 | F: TGTATCACCTCCTCCTCGTCTTCG | R: AATTAGCCAGTCATGCCGTCATCC |
| cluster-8388.71372 | F: CAGTGACAGCTTGTAGGACAGTGG | R: ACAACATCATCGGCGTCATCTTCG |
| cluster-8388.203329 | F: TGGATACTCTGTGCTGCCAACATG | R:CCAGGCAAGCGGCATTAGGTG |
| cluster-8388.168445 | F: GACGCTGGTCTTGGAATGGCTATG | R: GTACGATTGCGACATGGAGGAGTG |
| cluster-8388.64828 | F: GTCGGTGGTGGGTATGATGC | R: CCTGAGCTCCCACCAGGAAT |
| cluster-8388.299573 | F: TGATTCATGTTGCCAGCCTCCTTC | R:ATGCTCCACCGCTGTTGCTATTAG |
| cluster-8388.162261 | F: CAGAGGCTCCTTGTCAACGAAGTC | R: GAGGCATGGCAGAACAAGTCTCC |
| cluster-8388.157231 | F: CCTCGGTTCTTGGTCTGAAGTGTG | R: TGGAGGCTCTCGTTGGAAGTCTG |
| cluster-8388.172353 | F: TGTAGGCGGCGGCTGGTTAC | R: ACACCGTGTTAGATCATGCACCTG |
| cluster-8388.295361 | F: GGAAGCCTACTCAGCACATGGATC | R: CGTGAGAGGTGCAAGTATGGTCAG |
| cluster-8388.295722 | F: GAGCAAGTGGTAGTGGTAGGAAGC | R: CAACCAACCAACCAACCAACGAC |

Supplementary Table S2 Primers of qRT-PCR for validation of the seven DEGs involved in sesquiterpenoid and triterpenoid biosynthetic pathway

| Genes | Primers (5’→3′) |  |
| --- | --- | --- |
| *NDE* | F: ACAGTGTCAAGAGCGCGAAGAAC | R: CTGAAGATGGCGGCGAAGAAGG |
| *SE* | F: CCGCCTGGTTGTAGCAGTTCAC | R: ACCGTAGGATGGACGCAGAGTAC |
| *DS* | F: TGTCAACCTTGGACTTGAGCATCG | R: CGCAGATCATGGCAATTGGAACAC |
| *SHC* | F: GTGTTGGTGACTGGTGCTTCTGG | R: CAAGAGCAACGCCTTCGGATAGAG |
| *DS* | F: AGCGCCAAGAAGTCGAAGATGC | R: CTTCACCGCCGTCGTCACTG |
| *SPK* | F: TGCGTAAGCATCAGCAACGGTAG | R: TGTGATGCAGGAGAAGCTTGGATG |
| *SS* | F: CCGGACACAGAACTAAGGAGATCG | R: ACAAGCTACTAGCGCATCTACTGC |
