# Supplementary figures and images for: Comparative transcriptome analysis reveals sesquiterpenoid biosynthesis among 1-, 2- and 3-year old Atractylodes chinensis
Source: BMC Plant Biol. 2021 Jul 27;21:354. doi: 10.1186/s12870-021-03131-1 (PMC8314494; doi:10.1186/s12870-021-03131-1)

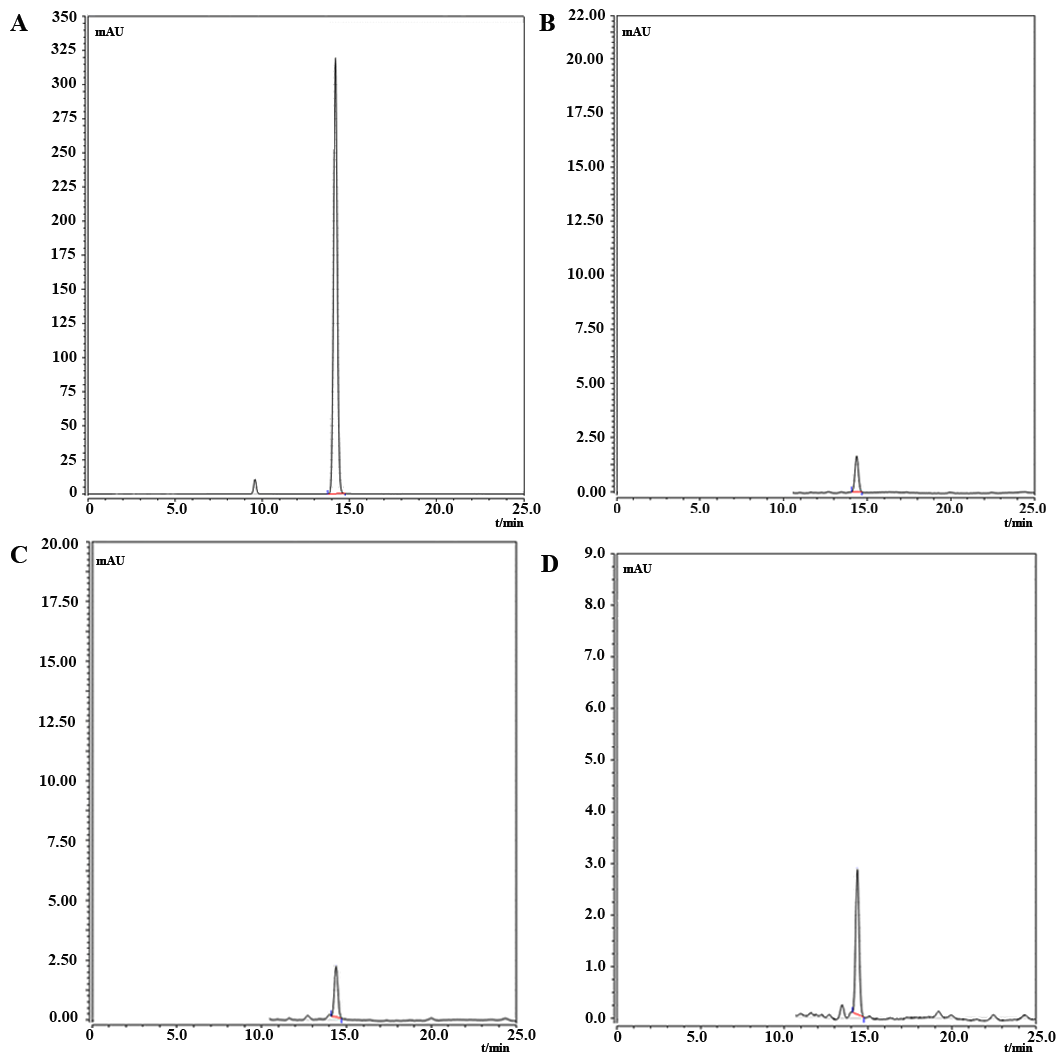

Supplement: Supplementary file 2 — Additional file 2: Fig. S1 The content of atractylodin in A. chinensis rhizomes. A. standard (20 mg∙ml−1); B. 1-year old rhizome; C. 2-year old rhizome; D. 3-year old rhizome. [file 12870_2021_3131_MOESM2_ESM.tif]
